# Legume Consumption and Colorectal Adenoma Risk: A Meta-Analysis of Observational Studies

**DOI:** 10.1371/journal.pone.0067335

**Published:** 2013-06-24

**Authors:** Yunqian Wang, Zhenhua Wang, Linna Fu, Yingxuan Chen, Jingyuan Fang

**Affiliations:** 1 Division of Gastroenterology and Hepatology, Renji Hospital, School of Medicine, Shanghai Jiao Tong University, Shanghai, People’s Republic of China; 2 Shanghai Institute of Digestive Disease, Shanghai, People’s Republic of China; 3 Key Laboratory of Gastroenterology & Hepatology, Ministry of Health, Shanghai Jiao Tong University, Shanghai, People's Republic of China; MOE Key Laboratory of Environment and Health, School of Public Health, Tongji Medical College, Huazhong University of Science and Technology, China

## Abstract

**Background:**

The anticancer effects of legumes have been explored extensively, but evidence from epidemiologic studies on colorectal adenoma is controversial. We performed a meta-analysis to assess these issues.

**Methods:**

A systemic search of several databases was conducted for relevant studies evaluating the relationship between legume intake and adenoma risk, with no language restriction, from January 1, 1966, to April 1, 2013.

**Results:**

Three cohort and eleven case control studies with 8,380 cases and a total of 101,856 participants were included in the analysis; the pooled odds ratio (95% confidence interval) for the highest vs. lowest consumption categories was 0.83 (0.75–0.93), with moderate level of heterogeneity (*I* ^2^ = 25.9% and *P* = 0.146) based on a random effects model. A decreased risk of adenoma was also observed in most of our subgroup meta-analyses.

**Conclusions:**

Higher intake of legumes significantly reduced the risk of colorectal adenoma in our meta-analysis. Nevertheless, due to possible confounders and bias, further investigations are warranted to confirm this relationship.

## Introduction

As the third most common cancer in males and the second in females, colorectal cancer (CRC) caused about 608,700 deaths in 2008, with over 1.2 million new cases diagnosed globally [1]. The occurrence and progression of the precursor lesion of CRC, colorectal adenoma (CRA), has attracted increasing attention over the past few decades. A better understanding of the environmental and genetic risk factors for CRA could improve our knowledge of the etiology of CRC and contribute to its primary prevention in high risk individuals. Dietary intervention has been proposed as a strategy to prevent and control colorectal tumorigenesis [2], and differences in the prevalence of certain cancers between different ethnic groups are partly attributed to dietary habits.

Regarded as “poor man’s meat”, legumes such as peas, beans, lentils, chickpeas and soybeans are not only rich in protein, but also significant sources of dietary fiber, resistant starch, folate, selenium, saponins, protease inhibitors, lectins, phytates and isoflavones with potential anticancer effects [3]. Legumes have played an important role in the traditional diets of Asia, South America and the Middle East for thousands of years, but their consumption is limited in western developed countries, where foods of animal origin constitute the staple diet [4].

Several epidemiologic studies have claimed that a high intake of legumes is associated with a significantly decreased risk of CRC [5], [6], [7], but the results of other studies are inconsistent [8], [9], [10], [11]. Despite their various antitumor constituents, limited evidence for a protective role of legumes against CRC was found by the World Cancer Research Fund/American Institute of Cancer Research (continuous update project report, 2011) after a thorough review of the relevant epidemiologic literature [12]. Animal studies have yielded fairly consistent results that a soy based diet or isoflavones inhibit the formation of aberrant crypt foci, a well accepted precursor of CRC and CRA, but there are no clear conclusions regarding the occurrence of chemically induced CRC [13]. In a clinical study using microarray technology, it was observed that increased intake of vegetables (including legumes) resulted in down-regulation of genes promoting cell proliferation and bioactivation of procarcinogens and up-regulation of genes involved in cell growth arrest in normal intestinal mucosa from both adenoma patients and healthy controls. Furthermore, the authors found that, in patients with CRA, the genes modulated by vegetable intake were responsible for relatively later stages of the evolution of colorectal neoplasms, whereas the genes modulated in healthy controls were involved in the initial phase [14], indicating that the protective effect of legumes might be significant in the earlier stages of colorectal carcinogenesis.

A growing body of epidemiologic literature has assessed the association between vegetable consumption and CRA, with many studies evaluating the potential protective effect of legumes. However, the sample sizes of most studies have been too small to reflect the relationship precisely. Additionally, meta-analysis after synthesizing the available evidence may provide a more reliable and conservative result than any single studies on associations between food ingredients and cancer risk [15]. Therefore, we conducted a meta-analysis to summarize quantitatively the available evidence and reach a consistent conclusion with respect to whether an association exists between higher legume consumption and lower CRA risk.

## Materials and Methods

### Identification of Studies

We made a systemic search of The Cochrane Library, MEDLINE and Embase bibliographic databases between January 1, 1966 and April 1, 2013, seeking all published articles including the following medical subject headings or key words: 1) soy OR soybeans OR beans OR peas OR legumes OR tofu OR soymilk OR miso OR natto OR lentils OR vegetable; 2) adenomas OR polyps OR adenomatous polyps OR cancer OR tumor OR carcinoma OR neoplasm; in combination with 3) colon OR rectum OR colorectal OR large bowel. There was no language limitation. We expanded our search strategy to diet or food and CRA to guarantee that relative articles that did not contain the aforementioned terms in their abstract could be identified. A manual search of the references cited in all of the obtained literature was also conducted to identify additional articles. Two assessors (Yunqian Wang and Zhenhua Wang.) independently investigated all papers considered for inclusion; any disagreements were solved by a third reviewer (Yingxuan Chen). We conducted this meta-analysis following the Meta-analysis of Observational Studies in Epidemiology guidelines [16].

### Inclusion and Exclusion Criteria

Studies were included if they met the criteria as follows: 1) case control or cohort studies published as original articles; 2) provided legume consumption categories for adenoma cases and non-cases groups; 3) adjusted relative risks (RRs) or odds ratios (ORs) with corresponding 95% confidence intervals (CIs), or the data necessary to calculate these, were reported. Animal studies, *in vitro* researches, case reports, ecological studies and reviews were not considered eligible. Given that sex, age of patient and the number or size of prior adenomas are the primary factors associated with adenoma recurrence [17], studies focusing on the recurrence or growth of adenoma were not considered in our present analysis. We also abandoned studies that included patients with ulcerative colitis, Crohn’s disease or familial adenomatous polyposis or who had undergone colectomy. When a study seemed to have been published in duplicate, we selected the version containing the most comprehensive information.

### Data Extraction

The data abstracted from each study included the last name of the first author, the year of publication, the study population, the numbers of case and control participants, risk estimates (highest vs. lowest intake) with their corresponding 95% CIs, and adjustment factors. Potential sources of heterogeneity, such as type of subjects, design of study, definition of exposure (assessment methods), and procedures for colorectal examination were also extracted and analyzed in the sub-group analyses. Most of the studies assessed CRA risk with respect to total legume consumption. When more than one type of legume was evaluated, we selected the most representative. This measurement was prioritized in a descending order of total legume, a certain type of legume or its product. If separate risk estimates for males and females were available in one study, we treated it as two separate studies. When a study provided several risk estimates, we chose the maximally adjusted models. If data for total adenomas and other types of adenomas (i.e. high or low risk adenomas) were both presented, we selected the former, which comprised more cases.

### Statistical Analysis

The meta-analysis was performed by combining the adjusted ORs or RRs of the highest compared with the lowest legume consumption level based on random effects model using DerSimonian-Laird method, which incorporated both within and between study variability. To evaluate the weighting of each study, the standard error for the logarithm OR of each study was calculated and regarded as the estimated variance of the logarithm OR [18]. Random effects model with restricted maximum likelihood estimate, which is more appropriate if the number of included studies is small, was also used to confirm the final risk estimates [19]. Heterogeneity was estimated by the Cochrane *Q*-test together with the *I* ^2^ statistic. A two-sided *P* value <0.1 or an *I* ^2^ value >50% indicates substantial heterogeneity across studies [20]. Sensitivity analysis was conducted by excluding each study in turn to evaluate the stability of the results. Begg’s funnel plots were constructed [21], and checked by Egger’s regression method. Any asymmetry observed or *P*<0.05 indicated potential publication bias [22]. Given that the prevalence of CRA was relatively low, we ignored the distinction between ORs and RRs, and regarded RRs from cohort studies as ORs for the purpose of calculations [23]. All analyses were conducted using STATS (version 10.0; College Station, TX, USA) and SAS software (version 9.1 SAS Institute., Cary, N.C., USA).

## Results

### Study Characteristics

According to the above search strategy, 1726 potential suitable articles were initially identified. After further assessment by reading the titles and abstracts, 109 publications might fulfill the inclusion criteria were reviewed in full. Of these, 97 articles were excluded, and the reasons as follows: outcome as recurrence of adenomas (n = 26) or colon epithelial cell proliferation (n = 2), exposures other than legume (n = 31), dietary pattern and CRA risk (n = 12), dietary factors and polyps plasma marker (n = 7), duplicate publication (n = 4), gene variation without assessing legume(n = 13), no useful data (p value only, n = 2). Another two studies were identified by systematic reference review [24], [25]. Figure S1 summaries the process of identifying and selecting of relevant studies. Ultimately, 14 studies with 8,380 cases and a total of 101,856 subjects were included in the meta-analysis. Six were hospital based case control studies [26], [27], [28], [29], [30], [31], five were population based case control studies [24], [25], [32], [33], [34], and the remainder were cohort designs [35], [36], [37]. Eight of the 14 studies involved US populations [27], [28], [30], [33], [34], [35], [36], [37]; one was from Europe [32]; the other five were from Asia, with three from Japan [24], [25], [26], one from Korea [29], and one in Malaysia [31], respectively. Only three studies presented separate data for males and females [25], [28], [29], two included males only [24], [35], and one was conducted with females only [36]. The number of cases and controls ranged from 53 to 3057 and 59 to 29,413, respectively. The cohort sizes ranged from 2,818 to 16,448. In all studies, cases were patients with newly diagnosed CRA ascertained by endoscopy and histology. Diets were assessed by validated or non-validated food frequency questionnaires (FFQs) with very different length of foods list (from25 to 276 food items). Risk estimates were below one in most studies included, but only three studies and one subgroup analysis in Kato’s study found a statistically significant inverse relationship between legume intake and adenoma risk [26], [31], [33], [36]. More detailed characteristics of the included studies, which were published between 1990 and 2011, are summarized in Table S1.

### Meta-analysis Results

**Figure 1:**
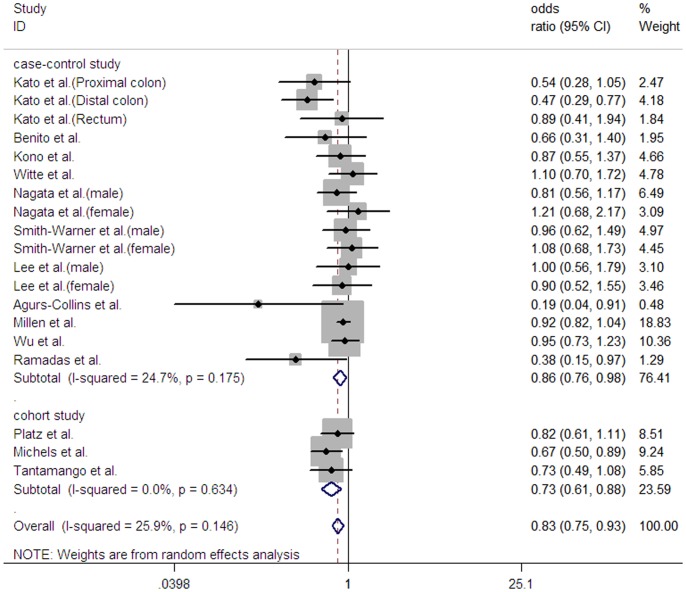
Forest plot of legume consumption (highest vs. lowest category) and colorectal adenoma risk. The square represents the point estimate of each study and the size is proportional to its weight in the meta-analysis. The horizontal line through the square represents its 95% confidence interval. The diamond indicates the pooled risk ratio of the analysis; the left and right vertices of the diamond reflect the 95% confidence interval.

With slight evidence of heterogeneity (*I* ^2^ = 25.9% and *P* = 0.146), the pooled OR with its 95% CI for the highest compared with the lowest consumption of legumes was 0.83 (0.75–0.93) based on the random effects model with DerSimonian–Laird method (Figure 1), and 0.84 (0.75–0.94) with maximum likelihood estimate, suggesting that higher consumption of legumes was associated with a statistically significant 17% decreased risk of CRA.

### Subgroup Analyses

As an essential part of the meta-analysis, stratified analyses were applied to investigate and identify underlying sources of heterogeneity (Table 1). When stratified by geographic region, studies conducted in Asia showed a slightly more pronounced inverse correlation between legume consumption and adenoma risk (*P* for heterogeneity = 0.197, *I* ^2^ = 27.9%, summary RR (SRR) = 0.77, 95% CI = 0.65–0.93) than those from western countries (*P* for heterogeneity = 0.239, *I* ^2^ = 22.1%, SRR = 0.88, 95% CI = 0.81–0.96, *P* for difference = 0.202). On stratified analysis by gender, the SRRs for CRA risk according to legume intake were 0.86 for males (95% CI = 0.70–1.01, *n* = 5) and 0.76 for females (95% CI = 0.60–0.93, *n* = 4). The difference between gender strata was not meaningful (*P* for difference = 0.434). Regarding type of study, heterogeneity was attenuated in cohort studies and the SRR showed a significantly lower risk of adenoma (*P* for heterogeneity = 0.634, *I* ^2^ = 0.0%, SRR = 0.73, 95% CI = 0.61–0.88) compared with the case control studies (*P* for heterogeneity = 0.175, *I* ^2^ = 24.7%, SRR = 0.86, 95% CI = 0.76–0.98, *P* for difference = 0.063).

**Table 1:** Stratified analyses of pooled risk estimates with 95% confidence intervals (CIs) for the highest compared with the lowest legume intake and the colorectal adenoma risk.

factor	subgroup	No. ofstudies	Pooled estimate(95% CI)	Heterogeneity		Publication bias	
				p	I^2^ (%)	Begg’s test	Egger’s test
Populations	Asian	5	0.77(0.65–0.93)	0.197	27.9	1.000	0.583
	Western	9	0.88(0.81–0.96)	0.239	22.1	0.474	0.222
Gender	Male	5	0.86(0.70–1.01)	0.952	0.0	0.086	0.073
	Female	4	0.76(0.60–0.93)	0.274	22.8	0.308	0.080
Study designs	Case-control	11	0.86(0.76–0.98)	0.175	24.7	0.065	0.092
	Cohort	3	0.73(0.61–0.88)	0.634	0.0	1.000	0.900
Type of subject	Symptomatic#	6	0.87(0.75–1.01)	0.128	35	0.074	0.181
	Asymptomatic*	8	0.87 (0.78–0.94)	0.237	23.2	0.466	0.106
FFQ types	Validated	7	0.84(0.74–0.96)	0.200	28.6	0.386	0.198
	Not validated	7	0.83(0.71–0.98)	0.162	30.1	0.062	0.078
Colorectal examination	Sigmoidoscopy	4	0.89(0.80–0.99)	0.172	39.9	0.734	0.790
	Colonoscopy	10	0.81(0.70–0.94)	0.185	24.3	0.837	0.315
Adjustment	BMI	10	0.89(0.81–0.96)	0.481	0.0	0.837	0.449
	Alcohol	9	0.88(0.76–0.98)	0.191	26.5	0.755	0.187
	Smoking	11	0.89(0.82–0.97)	0.328	11.4	1.000	0.323
	Energy intake	8	0.87(0.77–0.98)	0.398	4.8	0.350	0.770
	NSAID	6	0.89(0.81–0.97)	0.164	34.6	0.548	0.289
	Exercise	8	0.87(0.80–0.96)	0.075	45.6	0.108	0.068

# Patients from hospital based case control studies.

* Participants from cohort studies and population based case control studies were regarded as asymptomatic subjects (with no signs including diarrhea, bloating, abdominal pain, and fecal occult blood). Abbreviations: FFQ: food frequency questionnaire; BMI, body mass index; NSAID, non-steroidal anti-inflammatory drug.

A similar inverse correlation was found when the ORs of colonoscopy-based studies were combined, whereas the sigmoidoscopy- based studies showed a relatively weak protective effect. When stratified by dietary assessment methods, there was no difference between studies using validated FFQs and non-validated ones.

Detection rates of polyps or advanced adenomas were different in symptomatic patient group compared with asymptomatic screening participants [38]. A significant negative relationship was found for those studies with asymptomatic participants. However, only a borderline significant association was observed in symptomatic patients, and the stratified analysis did not show absence/presence of symptoms was the source of heterogeneity.

High legume consumption may be interrelated with a healthful diet or lifestyle (i.e. daily exercise, no smoking and low intake of alcohol). Moreover, Body mass index (BMI) and use of non-steroidal anti-inflammatory drugs (NSAIDs) are the potential confounders of CRA risk. When we restricted the meta-analysis to ten studies that reported OR adjusted for BMI, a significant tendency for higher legume consumption to reduce risk of CRA was found (SRR = 0.89, 95% CI = 0.81–0.96, *P* for heterogeneity = 0.481, *I* ^2^ = 0.0%). Similar results were obtained by analyses controlled for smoking (SRR = 0.89, 95% CI = 0.82–0.97, *P* for heterogeneity = 0.328, *I* ^2^ = 11.4%), alcohol (SRR = 0.88, 95% CI = 0.76–0.98, *P* for heterogeneity = 0.191, *I* ^2^ = 26.5%), NSAID use (SRR = 0.89, 95% CI = 0.81–0.97, *P* for heterogeneity = 0.164, *I* ^2^ = 34.6%) and exercise (SRR = 0.87, 95% CI = 0.80–0.96, *P* for heterogeneity = 0.075, *I* ^2^ = 45.6%). Total energy intake is an important confounder in epidemiological research assessing the association between diet or nutrition and chronic diseases [39]. When we pooled data of the 8 studies adjusted for energy intake, the result did not substantial changed (SRR = 0.87, 95% CI = 0.77–0.98, *P* for heterogeneity = 0.398, *I* ^2^ = 4.8% ).

### Sensitivity Analysis and Publication Bias

**Figure 2:**
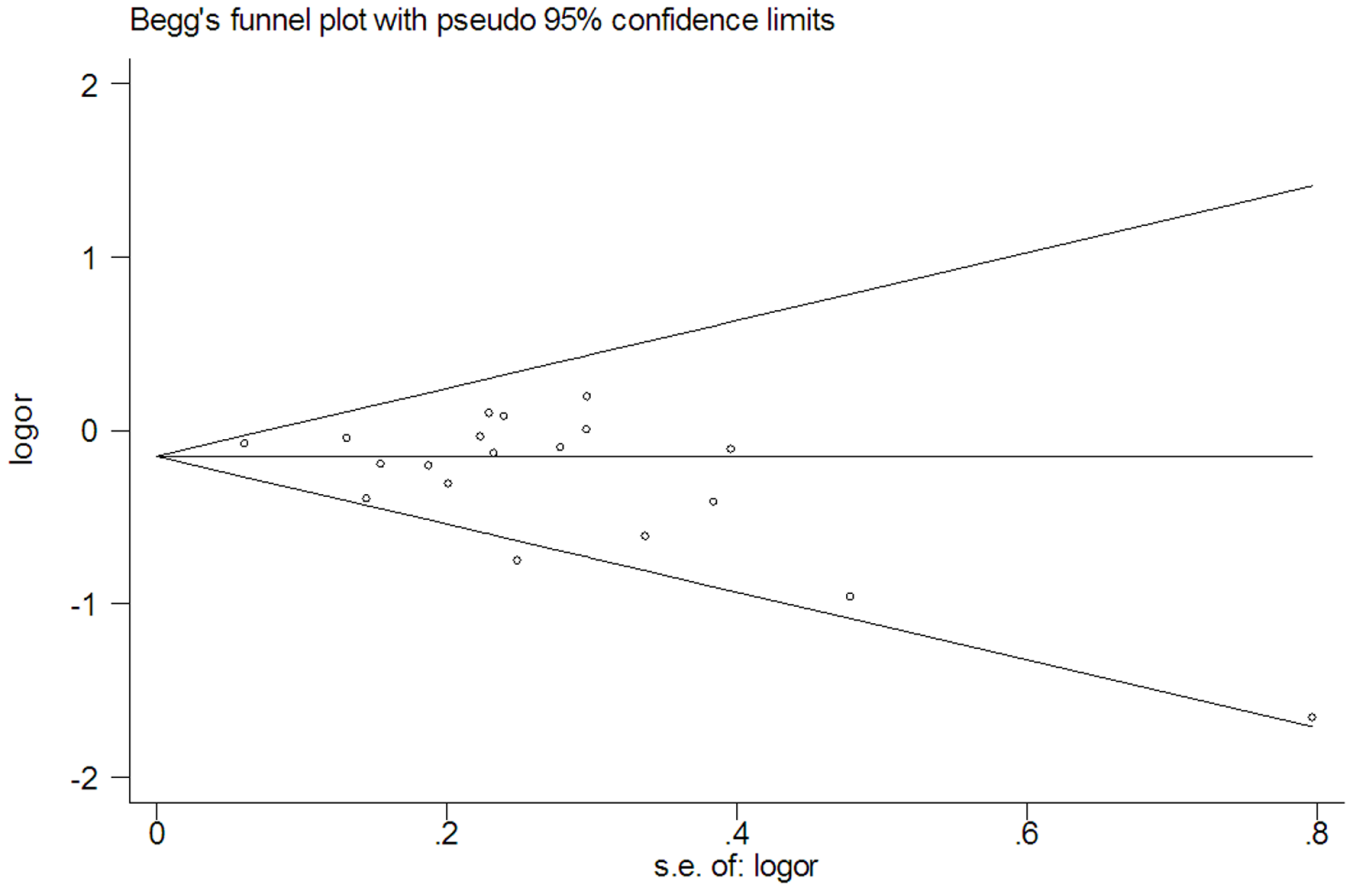
Begg’s funnel plot of the included studies.

When each study was excluded from the meta-analysis in turn, the summary OR did not change fundamentally, indicating that our results could not be solely attributed to the effect of a single study (Figure S2). In addition, no evidence of funnel plot asymmetry was observed (Figure 2). Neither Begg’s rank correlation (*P* = 0.208) nor Egger’s weighted regression method (*P* = 0.076) showed any publication bias.

## Discussion

This is the first meta-analysis to combine comprehensively the available epidemiologic studies on the relationship between legume consumption and CRA risk. Based on the data extracted from three cohort studies and eleven case control studies – which were generally well organized and controlled for various confounders – and subgroup analyses together with assessments of publication bias and sensitivity, the conclusion can be drawn that a diet containing greater amounts of legumes is associated with a lower risk of CRA.

Due to the great variety of anticarcinogens in legumes and their potential synergistic and additive actions, the mechanism involved in the chemoprotective effect of legumes against CRA might be complex. Non-digestible carbohydrates, including fiber and resistant starch, are abundant in legumes [40] and act as substrates for anaerobic fermentation by colonic bacteria in the large bowel, resulting in the production of short chain fatty acids (SCFAs) [41]. Butyrate, a major SCFA, has been shown to be protective by inhibiting histone deacetylase and thereby modulating the expression of genes involved in cancer cell proliferation, differentiation and apoptosis *in vitro* [42], [43] and *in vivo* [44]. Other well recognized antitumor constituents of legumes are flavonols and isoflavonols. An epidemiologic study suggested that a flavonol rich diet was associated with reduced risk of recurrence of advanced adenoma [45]. A previous meta-analysis showed an approximately 16% decrease in CRC risk associated with consumption of isoflavone (combined risk estimate = 0.84, 95% CI = 0.72–0.98) [46]. Bowman–Birk inhibitors extracted from legumes have already received approval for human trials from the US Food and Drug Administration and have been demonstrated to play important roles in several biologic processes related to the development of CRC, including inflammatory disorders, cell growth regulation/dysregulation and angiogenesis [47], [48]. Micronutrients derived from legumes such as folate, selenium and other bioactive phytochemicals, including saponins, phytic acid, lectins and phytosterols, are promising as agents against cancer [49].

It is now increasingly acknowledging that in addition to environment factors, common genetic variation might affect susceptibility to risk factors by altering the rates of activation and detoxification of environmental carcinogens [50]. A good example of this relationship is the potential interaction between transforming growth factor beta (TGF β) genetic polymorphisms, smoking, and CRC risk [51]. Another article confirmed the important role for single nucleotide polymorphisms (SNPs) as a contribution to CRC [52], also indicating that gene-environment interactions in carcinogenesis should be taken into consideration in the future.

Several limitations of our meta-analysis should be taken into account. First, eleven of the 14 included studies had case control designs, with the inevitable disadvantage of recall bias or selection bias. On stratified analysis, heterogeneity due to the case control subgroup might have contributed to the total heterogeneity, and cohort studies may more accurately reflect the true situation. Secondly, errors in the measurement of exposure and outcome might be responsible for discrepant results in sub group analyses. As for exposure evaluation, notable variations ranging from measurement of consumption categories to the food items consumed were observed across the included studies, studies conducted in eastern Asia, where soybeans are popular, contained more soy based legume food items than studies in western countries, where beans, peas and lentils are the most commonly consumed members of the Leguminosae and consumption tends to be lower. This may account for the differences observed in pooled estimated risks stratified by geographic region. As for different procedures for colorectal examination, sigmoidoscopy based studies may misclassified patients with adenomas in proximal colon as controls, which could result in underestimating the protective effects of legume against CRA. Besides, the fundamental objective of most published studies is not to determine the particular relationship between legume intake and CRA risk, and the limited literature did not allow us to investigate the protective effects of different members of the Leguminosae family. Finally, despite the extensive search we made in three databases and no suggestion of major publication bias in formal assessments performed, we could not completely deny the unpublished null results exists.

In conclusion, our results support the hypothesis that higher consumption of legumes is related to a reduction in the prevalence of CRA. Considering the widespread cultivation of legumes and the high morbidity and mortality of CRC, recommendations concerning increased consumption of legume based foods in everyday life might be a highly cost effective approach to CRC prevention via decreasing the incidence of CRA. Nevertheless, more large scale prospective cohort studies with appropriate dietary measurement methods or clinical intervention trials are needed to confirm this protective role of legumes against precancerous lesion formation in the large bowel.

## Supporting Information

Figure S1 **Flow diagram of the relevant study selection process.**
(TIF)

Figure S2 **Sensitivity analysis of studies of legume consumption and colorectal adenoma risk.**
(TIF)

Table S1 Characteristics of studies of colorectal adenoma and legume consumption.
(DOC)

Table S2 PRISMA 2009 checklist.
(DOC)
