# Supplementary figures and images for: Legume Consumption and Colorectal Adenoma Risk: A Meta-Analysis of Observational Studies
Source: PLoS One. 2013 Jun 24;8(6):e67335. doi: 10.1371/journal.pone.0067335 (PMC3691186; doi:10.1371/journal.pone.0067335)

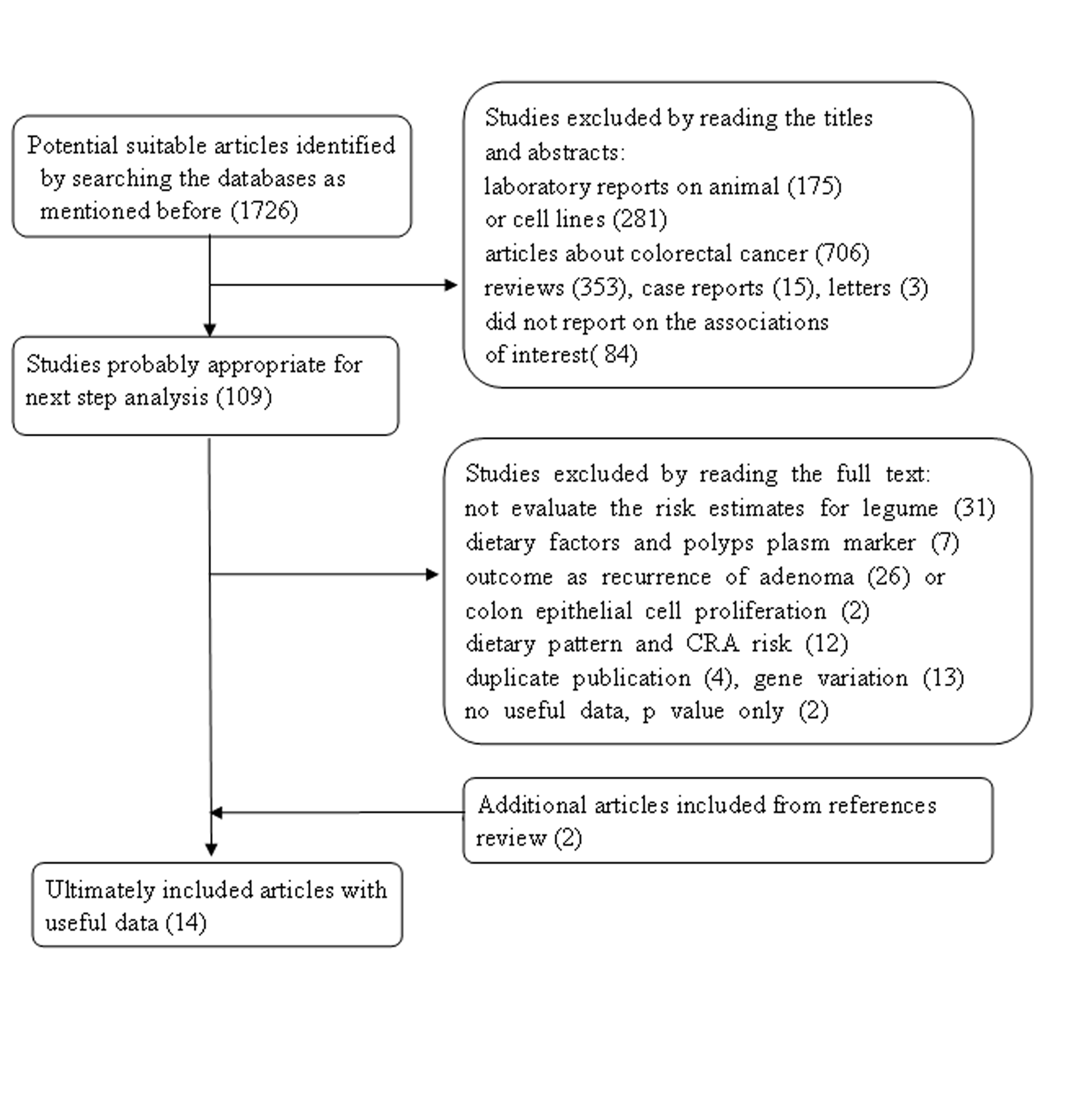

Supplement: Figure S1 — Flow diagram of the relevant study selection process. (TIF) [file pone.0067335.s001.tif]

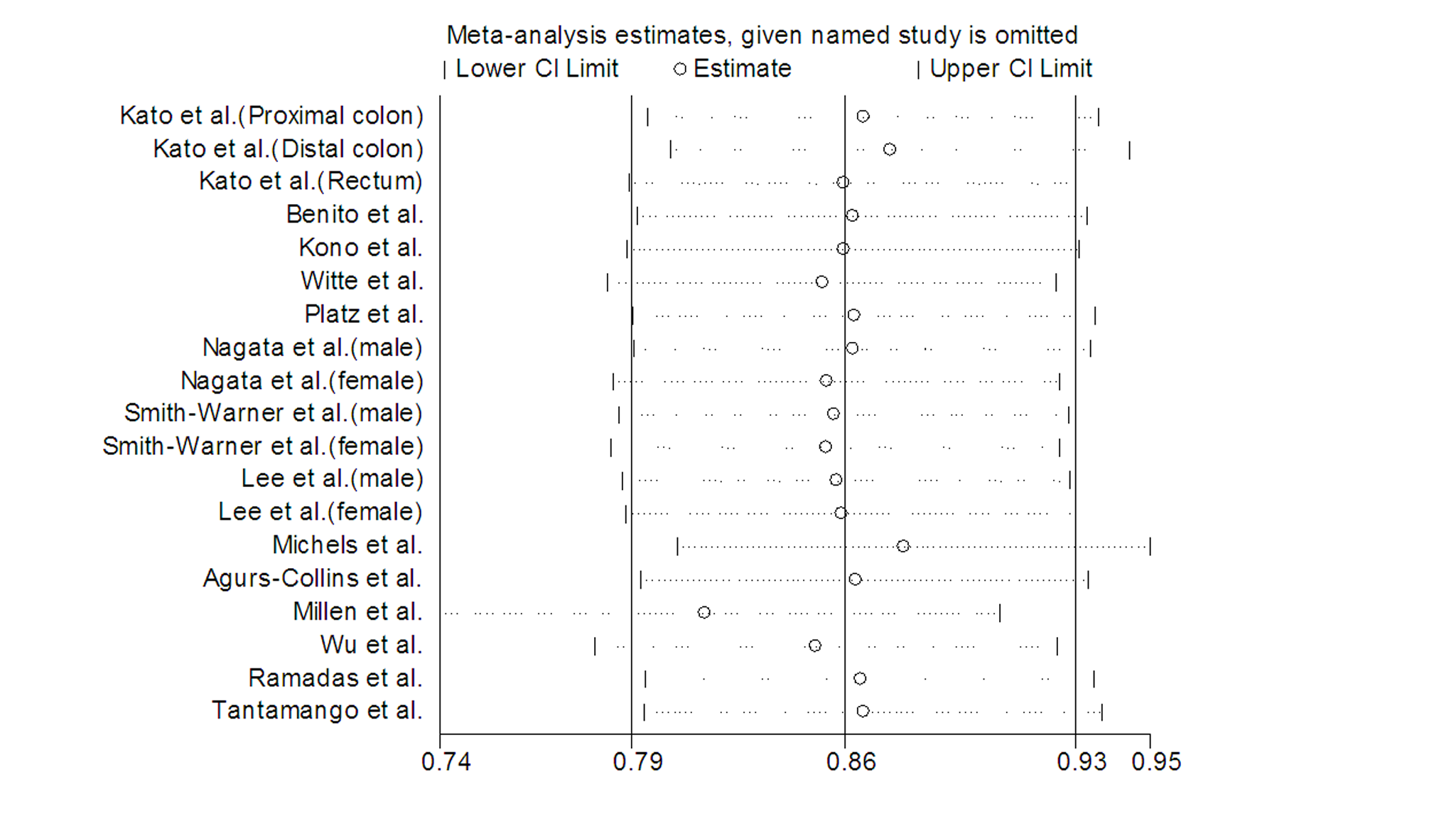

Supplement: Figure S2 — Sensitivity analysis of studies of legume consumption and colorectal adenoma risk. (TIF) [file pone.0067335.s002.tif]
