# Supplementary material for: Legume Consumption and Colorectal Adenoma Risk: A Meta-Analysis of Observational Studies
Source: PLoS One. 2013 Jun 24;8(6):e67335. doi: 10.1371/journal.pone.0067335 (PMC3691186; doi:10.1371/journal.pone.0067335)
Supplement: Table S1 — Characteristics of studies of colorectal adenoma and legume consumption. (DOC) [file pone.0067335.s003.doc]

**Table S1. Characteristics of studies of colorectal adenoma and** legume consumption

| **Reference** | **Country,**  **Study design** | **Colorectal examination** | **Cases/controls**  **(cohort size)** | **Sex** | **Exposure definition** | **Consumption**  **categories**  **(highest vs. lowest)** | **OR/RR(95%CI),**  **(assessment)** | **Adjustment factors** |
| --- | --- | --- | --- | --- | --- | --- | --- | --- |
| Kato  1990(26) | Japan,  HCC | Col | 525/578 | both | beans | 1-2 times/month vs.  daily | PC :0.54(0.28-1.05)  DC :0.4 (0.29-0.77)  R : 0.89 (0.41-1.94)  (questionnaire, 25 foods) | age, sex and residence |
| Benito,  1993(32) | Spain,  PCC | 80% Col | 101/242 | both | Legumes | <1vs.>6times/month | 0.66 (0.31-1.40)^#^  (99-item SFFQ) | age, sex, physical activity in longest held job, and rural residence |
| Kono,  1993(24) | Japan,  PCC | Sig | 187/1,557 | M | soy paste soup | <1 vs.≥2 bowls/day | 0.87 (0.55-1.37)  (questionnaire) | age, smoking, alcohol and BMI |
| Witte  1996(27) | USA,  HCC | Sig | 488/488 | both | legumes | 0.5vs.8.5servings/week | 1.1 (0.7-1.72)  (126-item SFFQ) | age, sex, race, BMI, exercise,  smoking, calories, fat intake, dietary  fiber, fotate, multivitamin use |
| Platz  1997(35) | USA  Cohort | Col | 690/16,448 | M | legumes | 0.1 vs. 2.6 g/day | 0.82 (0.6-1.1)  (131-item SFFQ, validated) | age, FHCC, BMI, smoking,  multivitamin use, exercise, NSAIDs  use, energy intake, alcohol, red meat  intake folate and methionine |
| Nagata  2001(25) | Japan,  PCC | Col | M181/12,607  F:98/15,754 | both | soy | M: 40.6 vs.166.4g/day  F: 38.5 vs.148.6g/day | M:0.81 (0.56-1.17)  F:1.21 (0.68-2.18)  (169-item SFFQ, validated) | age, energy intake, smoking, and alcohol |
| Smith-Warner  2002(28) | USA,  HCC | Col | 564/682 | both | legumes | M:1vs.5servings/day  F:1.1vs.5.5servings/day | M:0.96 (0.62-1.49)  F: 1.08 (0.68-1.74)  (153-item SFFQ) | age, energy, fat intake, BMI, smoking,alcohol, NSAIDs use, multivitamin useand hormone replacement therapy |
| Lee  2005(29) | Korea,  HCC | Col | 539/2,576 | both | soybeans | M:quintile1vs.quintile5  F:quintile1vs.quintile5 | M:1.00 (0.56-1.79)  F: 0.90 (0.52-1.55)  (questionnaire and  interview ,55 foods) | age, smoking, BMI, education, and  energy intake |
|  |  |  |  |  |  |  |  | ***(Continued)*** |

***Table S1. (Continued)***

| **Reference** | **Country,**  **Study design** | **Colorectal examination** | **Cases/controls**  **(cohort size)** | **Sex** | **Exposure definition** | **Consumption**  **categories**  **(highest vs. lowest)** | **OR/RR(95%CI)**  **(assessment)** | **Adjustment factors** |
| --- | --- | --- | --- | --- | --- | --- | --- | --- |
| Michels  2006(36) | USA,  Cohort | Sig | 633/10,368 | F | legumes | <3 vs.>7 servings/day | 0.67 (0.51-0.90)  (116-item SFFQ, validated) | age, FHCC, BMI, exercise, NSAID  use, alcohol, smoking, energy intake,  red meat consumption, calcium intake and hormone replacement therapy |
| Agurs-Collins  2006(33) | USA,  PCC | Col | 53/133 | both | legumes | <1 vs. ≥3 times/week | 0.19 ( 0.04-0.91)  (39-item FFQ) | age, sex, smoking, alcohol, weight, aspirin, exercise, FHCC |
| Millen  2007(34) | USA,  PCC | Sig | 3057/29,413 | both | dry beans | quintile1 vs. quintile5  (medians : 0.05 vs. 0.4 pyramid servings/day) | 0.92 (0.81-1.03)  (137-item SFFQ, validated) | age, sex, race, study center, education, FHCC, smoking, alcohol, NSAIDs  use, exercise, BMI and hormone  replacement therapy |
| Wu  2009(30) | USA,  HCC | Col | 764/1,517 | both | legumes | tertile 1 vs. tertile 3  (servings/week) | 0.95 (0.74-1.24)  (108-item SFFQ, validated) | age, sex, race, BMI, smoking, alcohol, NSAID use, exercise, FHCC and red meat intake, energy intake, and  mutually adjusted for fruit intake |
| Ramadas  2009(31) | Malaysia,  HCC | Col | 59/59 | both | soybeansmiso or  tofu | <3 vs. ≥3 times/week | 0.38 (0.15-0.98)  (276-itemSFFQ, validated,  interview) | age, sex, race, physical activity, height, BMI, energy intake, smoking and alcohol |
| Tantamango  2011(37) | USA,  Cohort | Col | 441/2,818 | both | legumes | <1 time/month vs.  >3 times/week | 0.73 (0.49-1.08)  (55-item SFFQ, validated) | age, sex and BMI |

^#^ The article only provided adjusted OR. The 95% CI was estimated based on data available in the study.

Abbreviations: FFQ: food frequency questionnaire; SFFQ: semiquantitative food frequency questionnaire; BMI, body mass index; NSAID, non-steroidal anti-inflammatory drug; HCC, hospital based case control study; PCC, population based case control study; M: male; F: female; PC, proximal colon; DC, distal colon; R, rectum; FHCC, family history of colorectal cancer; Sig: 60-cm flexible sigmoidoscopy; Col: colonoscopy reaching cecum.
